# Severe Malaria Due to Plasmodium vivax With Pulmonary Involvement: A Case Report

**DOI:** 10.7759/cureus.102880

**Published:** 2026-02-03

**Authors:** Teresa Valido, Ana Goncalves, Marta Sanches, Teresa Costa Silva, Carlos Pereira

**Affiliations:** 1 Department of Internal Medicine, Unidade Local de Saúde de Amadora/Sintra, Amadora, PRT; 2 Department of Infectious Diseases, Hospital Curry Cabral, Lisbon, PRT; 3 Department of Internal Medicine, Hospital Beatriz Ângelo, Loures, PRT; 4 Intensive Care Unit, Hospital Beatriz Ângelo, Loures, PRT

**Keywords:** intensive care medicine, malaria, plasmodium vivax malaria, pulmonary malaria, severe malaria

## Abstract

Malaria is a disease caused by *Plasmodium* parasites and spread through the bites of female *Anopheles* mosquitoes. Although *Plasmodium falciparum* remains the primary species causing severe malaria, *Plasmodium vivax* is increasingly recognized, and its latent forms can reactivate months to years after exposure. A 46-year-old woman from India, who had traveled to Portugal for six months, was admitted to the emergency department with asthenia and fatigue for three days. On examination, she was febrile and hypoxemic. Additional evaluation revealed severe anemia, thrombocytopenia, and extensive areas of ground-glass opacification on chest tomography. She was admitted to the ICU and required high-flow oxygen therapy. During further evaluation, *P. vivax* was identified, with a parasitemia of 50% and a parasite density of 8,645 parasites/µL. Due to the unavailability of IV artesunate, treatment was initiated with artemether-lumefantrine and doxycycline, resulting in progressive improvement and successful oxygen weaning. By the third day of treatment, parasitemia had cleared. This case underscores the need for high clinical suspicion for *P. vivax* malaria in patients with relevant epidemiological exposure.

## Introduction

Malaria is a parasitic disease caused by the protozoan *Plasmodium*, transmitted through the bite of female *Anopheles *mosquitoes. The greatest mortality from malaria is associated with *Plasmodium falciparum *infection; however, malaria caused by non-falciparum species can also result in significant morbidity and mortality. *Plasmodium vivax *is the second most common form of malaria and the most geographically widespread, with more than two billion people at risk of infection [1,2]. WHO estimated that in 2024, 3.5% of malaria cases were due to *P. vivax *[3]. Malaria can lead to various life-threatening complications, including severe anemia, liver or renal impairment, cerebral malaria, and acute respiratory distress syndrome (ARDS) [4].

*P. vivax *has the capability to survive in a quiescent hepatic stage called the hypnozoite, which can reactivate months to years after the initial infection. In tropical areas, relapses occur in more than 80% of cases, with the first relapse usually within 30 days of initial symptoms. Subsequent relapses may occur several months or even years later [5].

The only drug available to prevent relapses is primaquine. WHO recommends additional treatment with primaquine at 0.5 mg/kg/day for at least seven days, which may improve adherence compared with the standard 14-day regimen, thereby reducing relapse rates [5]. The glucose-6-phosphate dehydrogenase (G6PD) status of patients should be assessed before primaquine use, as the drug can cause severe hemolytic anemia in patients with G6PD deficiency [5].

*P. vivax* infection mostly causes mild disease, with rare severe presentations ranging from 0.5% to 4.5% and a mortality rate of less than 1% [6,7]. WHO guidelines define severe malaria as the presence of at least one severity criterion, with no alternative diagnosis that better explains the presentation [5]. Criteria for severe malaria include reduced consciousness (Glasgow Coma Scale <11), prostration, recurrent seizures, metabolic acidosis, hypoglycemia, severe anemia (hemoglobin ≤7 g/dL or hematocrit ≤20%), acute kidney injury (serum/plasma creatinine >3 mg/dL or blood urea >20 mmol/L), jaundice (bilirubin >3 mg/dL), pulmonary edema or respiratory compromise (radiologic pulmonary edema or SpO₂ <92% on room air with respiratory rate >30/min, often with increased work of breathing and crackles on auscultation). Additional severe features include clinically significant bleeding (e.g., persistent or recurrent bleeding from mucosae or venipuncture sites, hematemesis, or melena), shock, and hyperparasitemia (*P. falciparum *>10%). Severe vivax malaria is defined using the same clinical and laboratory features, without mandatory parasite-density thresholds.

Respiratory dysfunction is one of the rarest manifestations of severe malaria due to *P. vivax*. A recent study in New Delhi, India, estimated that 3% of severe malaria cases can progress to ARDS [8]. Another study from Brazil identified female sex, comorbidities, poor respiratory status at hospital admission, and lower hemoglobin levels as risk factors for mortality due to *P. vivax* [9].

Treatment of severe vivax malaria should begin with full doses of parenteral antimalarial therapy (artemisinin derivatives or cinchona alkaloids) for at least 24 hours or until the patient can tolerate oral medication, followed by a full course of effective artemisinin-based combination therapy orally [5]. Patients presenting with acute pulmonary edema and severe hypoxemia may require positive end-expiratory pressure or continuous positive airway pressure [5].

This article was previously presented as a poster at the 22nd European Congress of Internal Medicine on March 7, 2024.

## Case presentation

A 46-year-old woman from New Delhi, India, who had been living in Portugal for six months, was admitted to the emergency department with asthenia, fatigue, and dizziness for the past three days. Her past medical history included hypertension and hypothyroidism, for which she was taking levothyroxine 0.1 mg daily and azilsartan 40 mg daily. The patient had no history of infectious diseases such as malaria and reported no recent travel other than her relocation from India six months earlier. She also denied cough, chest pain, or other symptoms.

On admission, she was pale, dehydrated, hypoxemic, and febrile. Despite these findings, she was hemodynamically stable, with a blood pressure of 111/62 mmHg and a heart rate of 70 bpm, and no other abnormal findings on physical examination.

**Figure 1:**
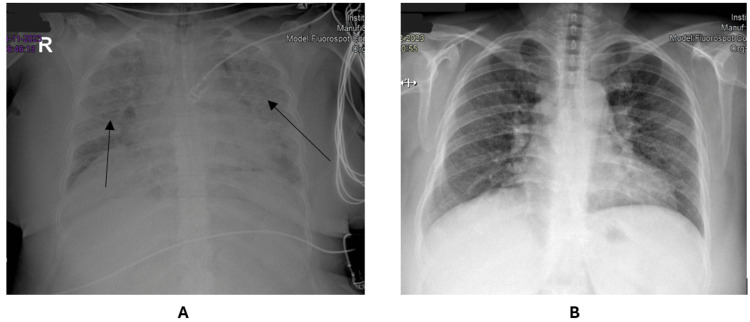
Chest X-ray at admission (A) and at discharge (B)

Further investigation revealed severe anemia with a hemoglobin level of 4.1 g/dL, microcytosis (mean corpuscular volume 74.4 fL), and hypochromia (mean corpuscular hemoglobin 19.6 pg). She also had thrombocytopenia (120,000 × 10⁹ platelets) but no coagulopathy or renal or hepatic dysfunction (Table 1). Chest X-ray showed bilateral infiltrates, suggestive of acute pulmonary edema (Figure 1A).

**Table 1:** Laboratory findings at admission ALT, alanine aminotransferase; aPTT, activated partial thromboplastin time; AST, aspartate aminotransferase; Hb, hemoglobin; INR, international normalized ratio; MCH, mean corpuscular hemoglobin; MCV, mean corpuscular volume

Finding	Result (as reported)	Adult reference range
Hb	4.1 g/dL	12.0-17.0 g/dL
MCV	74.4 fL	80-100 fL
MCH	19.6 pg	27-33 pg
Platelets	120 × 10⁹/L	150-400 × 10⁹/L
Coagulation (INR, aPTT)	0.9, 26.3 seconds	INR: 0.8-1.2; aPTT: 25-35 seconds
Serum creatinine	0.9 mg/dL	0.6-1.3 mg/dL
Hepatic function (AST/ALT, total bilirubin)	31 U/L, 24 U/L, 0.6 mg/dL	AST: 10-40 U/L; ALT: 7-56 U/L; total bilirubin: 0.2-1.2 mg/dL

**Figure 2:**
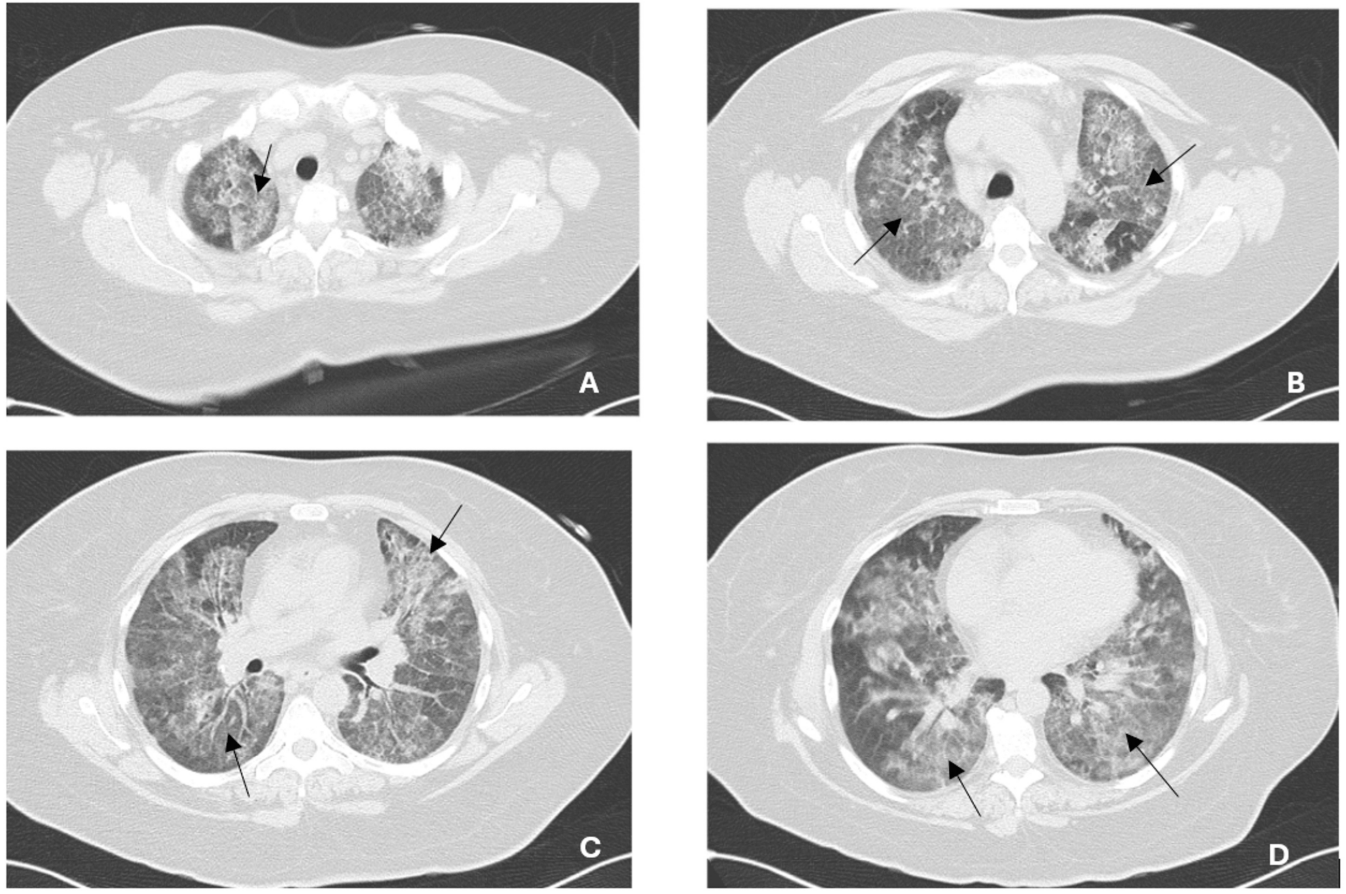
Thoracic CT scan (A, B) Pulmonary apices. (C, D) Lower pulmonary lobes. All images show extensive areas of ground-glass opacities.

A CT scan of the chest and abdomen was requested (Figure 2A-2D), which showed extensive areas of ground-glass opacities with a “crazy-paving” pattern, as well as hepatomegaly and spleen dimensions at the upper limit of normal. A vasculitic condition was initially suspected, and corticosteroid therapy was started (methylprednisolone 500 mg/day) while autoimmune studies were requested; these later returned negative.

On the second day of hospitalization, the patient experienced worsening hypoxemia, requiring oxygen therapy via a Venturi mask (VMK) with a fraction of inspired oxygen (FiO₂) of 60%. She was subsequently admitted to the ICU. Upon ICU admission, she was awake and oriented, with pale mucous membranes, tachycardia, and an increased respiratory rate. Arterial blood gas analysis revealed a partial pressure of oxygen (pO₂) of 67 mmHg on VMK 60%, prompting a switch to a high-flow nasal cannula at 45 L/min with FiO₂ of 80%.

**Figure 3:**
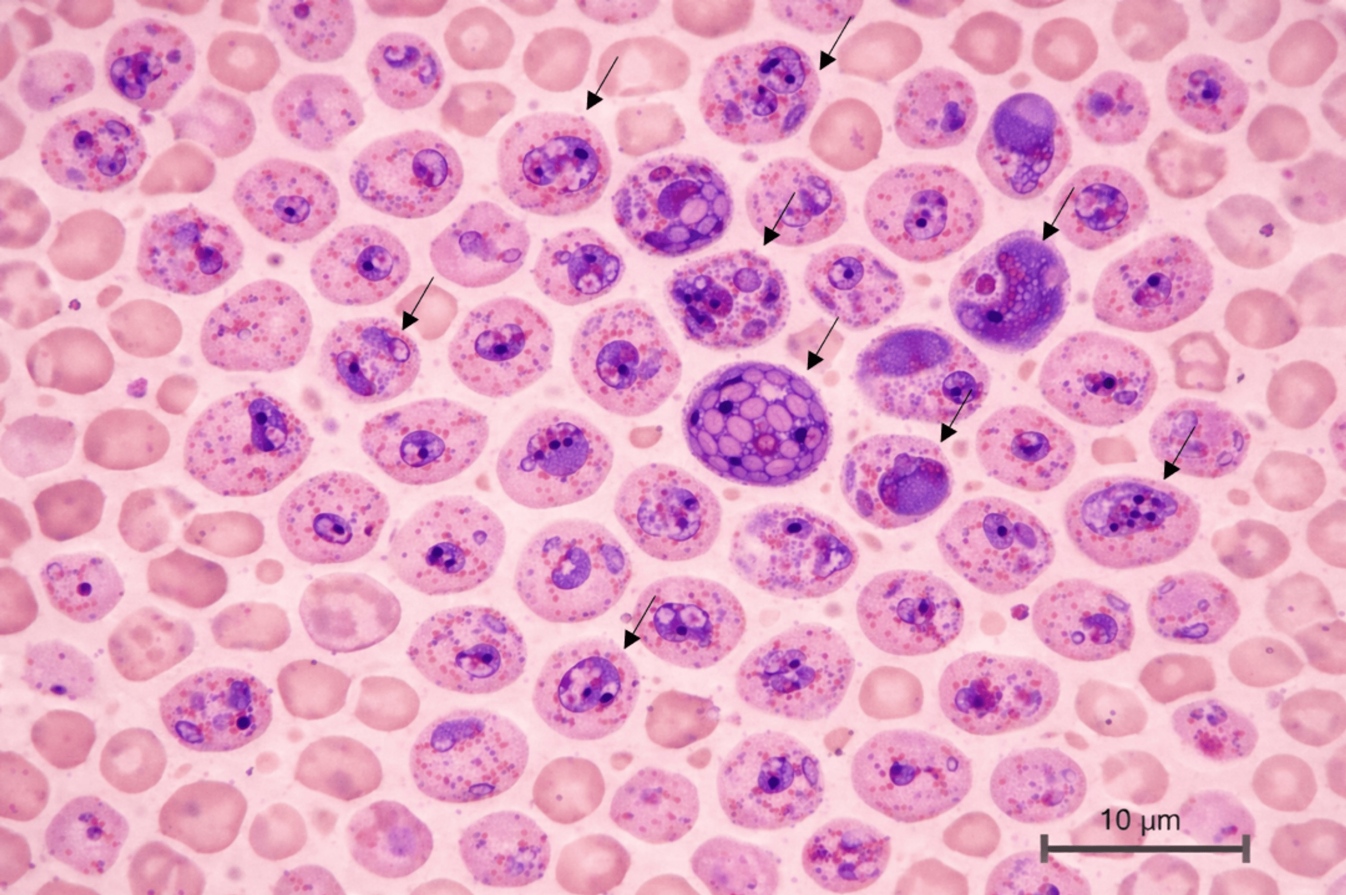
Peripheral blood thin smear revealing Plasmodium vivax Giemsa-stained thin blood film showing enlarged erythrocytes with Schüffner’s stippling and intraerythrocytic *P. vivax *trophozoites and schizonts (scale bar: 10 µm).

Given the epidemiological context, a *Plasmodium *investigation was requested. Results revealed a positive *P. vivax *antigen, and further evaluation with a peripheral blood thin smear demonstrated a parasitemia of 50% (Figure 3). All forms of the parasite life cycle were observed, with a density of 8,645 parasites/µL. Due to the unavailability of intravenous artesunate, therapy was initiated with oral artemether-lumefantrine 20 mg-120 mg (four tablets per dose at zero and eight hours, then four tablets twice daily for the next two days) and doxycycline 100 mg orally twice daily for seven days.

The patient showed progressive improvement, with oxygen therapy being tapered from the third day of treatment; parasitemia was negative on that day. She was transferred to the infectious diseases department, where her clinical course remained favorable. She was discharged after 10 days of hospitalization, breathing comfortably in ambient air, with a chest X-ray showing improvement of the previously observed infiltrates (Figure 1B).

Regarding anemia, hemolytic causes were ruled out based on normal haptoglobin, normal lactate dehydrogenase, and a negative Coombs test. Hematologic evaluation revealed vitamin B12 deficiency (156 pg/mL), with normal folate and iron levels. Intramuscular cyanocobalamin supplementation was initiated daily for one week, followed by twice-weekly dosing. The patient required transfusion of four units of red blood cell concentrate, with a hemoglobin level of 9 g/dL at discharge.

At discharge, the patient was awaiting G6PD assay results, which later returned normal. She subsequently completed a 14-day course of primaquine therapy for eradication of hypnozoites.

## Discussion

India has a tropical climate and remains endemic for both* P. vivax *and *P. falciparum*. Because malaria can mimic many other acute infections, any patient in whom malaria is clinically suspected should be tested without delay. Severe and complicated malaria is classically associated with *P. falciparum*; however, growing clinical experience and case reports indicate that *P. vivax,* once widely considered a relatively benign infection with low fatality, can also cause severe disease [10].

We describe a case of severe *P. vivax *malaria in an Indian patient with no prior documented malaria who had left an endemic area six months before hospital admission. The presentation was notable for profound anemia and acute lung injury, emphasizing the importance of travel history and epidemiological exposure in the clinical assessment.

Pulmonary involvement has been documented in severe vivax malaria, including non-cardiogenic pulmonary edema, ARDS, acute pulmonary injury, and interstitial pneumonitis [11-14]. In routine practice, these respiratory complications may be overlooked, potentially delaying diagnosis and the escalation of supportive care and increasing the risk of pulmonary edema from overly aggressive fluid administration.

Reported severe manifestations of *P. vivax *malaria most commonly include hepatic and renal dysfunction, severe anemia, ARDS, cerebral involvement, and multi-organ dysfunction [15]. *P. vivax *can trigger severe disease through a combination of host inflammatory responses and microvascular/endothelial dysfunction, even when peripheral parasitemia appears modest. Increasing evidence links severe vivax phenotypes to systemic inflammation with endothelial activation (e.g., elevated angiopoietin-2/ICAM-1 and related pathways), microvascular dysfunction, and endothelial glycocalyx degradation, which may promote capillary leak, impaired perfusion, and organ injury [16].

Timely recognition and initiation of appropriate antimalarial therapy can reduce complications and mortality and may also limit unnecessary exposure to broad-spectrum antibiotics. In this context, the availability of intravenous artesunate is pivotal, as current international guidelines recommend parenteral artesunate as the preferred initial therapy for severe malaria, irrespective of species [5]. Finally, relapse prevention is a critical component of vivax management; using a radical cure regimen to clear hypnozoites is essential to reduce recurrent episodes.

## Conclusions

This case highlights the growing recognition of *P. vivax *as a cause of severe, complicated malaria, a role traditionally associated with *P. falciparum*, as illustrated by a presentation with severe anemia and acute pulmonary injury, including imaging features such as ground-glass opacities and a crazy-paving pattern. Although respiratory complications, including acute pulmonary injury and ARDS, are increasingly reported, they likely remain underrecognized in clinical practice. The patient’s favorable outcome with prompt antimalarial therapy and supportive care underscores the importance of early identification and treatment.

This case also emphasizes the need to target the hypnozoite stage to prevent relapse, as supported by the successful completion of primaquine therapy. Furthermore, it highlights the importance of further research into parasite-host interactions to clarify the mechanisms underlying severe disease. Ultimately, improved clinician awareness and adherence to WHO-recommended management protocols are essential to reduce morbidity and mortality, particularly in non-endemic settings where *P. vivax *malaria may not be promptly considered.

## References

[1] Price RN, Commons RJ, Battle KE, Thriemer K, Mendis K (2020). Plasmodium vivax in the era of the shrinking P. falciparum map. Trends Parasitol.

[2] Battle KE, Lucas TC, Nguyen M (2019). Mapping the global endemicity and clinical burden of Plasmodium vivax, 2000-17: a spatial and temporal modelling study. Lancet.

[3] (2026). World malaria report 2025. https://www.who.int/teams/global-malaria-programme/reports/world-malaria-report-2025.

[4] Chang CY (2023). Clinical characteristics and outcome of severe malaria in Kapit, Sarawak, Malaysian Borneo. J Vector Borne Dis.

[5] (2026). WHO guidelines for malaria. https://www.who.int/publications/i/item/guidelines-for-malaria.

[6] Baird JK (2013). Evidence and implications of mortality associated with acute Plasmodium vivax malaria. Clin Microbiol Rev.

[7] Rahimi BA, Thakkinstian A, White NJ, Sirivichayakul C, Dondorp AM, Chokejindachai W (2014). Severe vivax malaria: a systematic review and meta-analysis of clinical studies since 1900. Malar J.

[8] Matlani M, Kojom LP, Mishra N, Dogra V, Singh V (2020). Severe vivax malaria trends in the last two years: a study from a tertiary care centre, Delhi, India. Ann Clin Microbiol Antimicrob.

[9] Val F, Machado K, Barbosa L (2017). Respiratory complications of Plasmodium vivax malaria: systematic review and meta-analysis. Am J Trop Med Hyg.

[10] Kochar DK, Das A, Kochar SK (2009). Severe Plasmodium vivax malaria: a report on serial cases from Bikaner in northwestern India. Am J Trop Med Hyg.

[11] Torres JR, Perez H, Postigo MM (1997). Acute non-cardiogenic lung injury in benign tertian malaria. Lancet.

[12] Munteis E, Mellibovsky L, Márquez MA, Mínguez S, Vázquez E, Díez A (1997). Pulmonary involvement in a case of Plasmodium vivax malaria. Chest.

[13] Pukrittayakamee S, Chantra A, Vanijanonta S, White NJ (1998). Pulmonary oedema in vivax malaria. Trans R Soc Trop Med Hyg.

[14] Price L, Planche T, Rayner C, Krishna S (2007). Acute respiratory distress syndrome in Plasmodium vivax malaria: case report and review of the literature. Trans R Soc Trop Med Hyg.

[15] Mehndiratta S, Rajeshwari K, Dubey AP (2013). Multiple-organ dysfunction in a case of Plasmodium vivax malaria. J Vector Borne Dis.

[16] Dayananda KK, Achur RN, Gowda DC (2018). Epidemiology, drug resistance, and pathophysiology of Plasmodium vivax malaria. J Vector Borne Dis.

